# Classification of Micro-Damage in Piezoelectric Ceramics Using Machine Learning of Ultrasound Signals

**DOI:** 10.3390/s19194216

**Published:** 2019-09-28

**Authors:** Gaurav Tripathi, Habib Anowarul, Krishna Agarwal, Dilip K. Prasad

**Affiliations:** 1 Department of Electronics Engineering, Indian Institute of Technology, Dhanbad 826004, India; gauravcr7rm.16je002566@ece.iitism.ac.in; 2 Department of Physics and Technology, UiT The Arctic University of Norway, 9037 Tromsø, Norway; anowarul.habib@uit.no (H.A.); krishna.agarwal@uit.no (K.A.); 3 Department of Computer Science, UiT The Arctic University of Norway, 9037 Tromsø, Norway

**Keywords:** structural health monitoring, ultrasound, feature design, classification

## Abstract

Ultrasound based structural health monitoring of piezoelectric material is challenging if a damage changes at a microscale over time. Classifying geometrically similar damages with a difference in diameter as small as 100 μm is difficult using conventional sensing and signal analysis approaches. Here, we use an unconventional ultrasound sensing approach that collects information of the entire bulk of the material and investigate the applicability of machine learning approaches for classifying such similar defects. Our results show that appropriate feature design combined with simple k-nearest neighbor classifier can provide up to 98% classification accuracy even though conventional features for time-series data and a variety of classifiers cannot achieve close to 70% accuracy. The newly proposed hybrid feature, which combines frequency domain information in the form of power spectral density and time domain information in the form of sign of slope change, is a suitable feature for achieving the best classification accuracy on this challenging problem.

## 1. Introduction

Damage detection is an important aspect for characterization of any material in structural health monitoring. It is of significant interest to detect the damage and its progression early, so that the possibility of structural failure can be determined with sufficient notice. A material may suffer micro-damages in harsh and extreme environmental conditions, such as corrosion, fatigue, and temperature fluctuations. Even with a new material sample unexposed to environment, microdefects may be present intrinsically. Operational stresses tend to concentrate along the periphery of the defects. In piezoelectric (PZT) ceramics, these microscale defects act as a precursor to damage that may limit the strength, lifetime, and performance of PZT ceramics as sensors. Moreover, in practical scenarios, it is important to identify the damage and its progression early in order to allow for failure prevention or failure preparedness. It can be of critical importance to human lives in applications such as in the health of a residential building or aeroplanes.

Additionally, detection of change in micro-damages is important for sensor technology for structural health monitoring. Prior to installation, all sensors must be calibrated and compensated for errors and noises arising from ambient conditions, including harsh operational environment and temperature fluctuations. Additional error in the measurement may be induced over time due to the minor defects in the sensor as it degrades with usage and age. This is often circumvented by correcting the acquired responses by a baseline compensation factor. Right after installation, the sensors are assumed to be healthy and thereafter considerable effort should be devoted for periodic assessment and monitoring of the health of the sensors [1]. However, a sensor with micro-scale defects exhibits higher-order effects and nonlinear piezoelectric behaviour that discourages the use of simple signal post-processing for correction. Therefore, it is important to detect, localize and quantify the defect in the piezo- ceramic in order to avoid false alarm in structural health monitoring applications.

Here we propose a methodology to quantify the precursor to damages in the PZT ceramic sensors that can provide sufficient warning time for early retrofitting of the structure. The precursor to damages is defined as any hidden feature resulting from early degradation of the structure or the sensor, leading to microscale defects or microstructural and morphological changes. Damage detection in PZT sensor has two advantages in validation of our proposed technique: (a) acting as a calibrating benchmark sensor whose health reflects the damage in the structure; and (b) acting as a master sensor that tracks the health of the structure continuously.

Ultrasonic inspection is used extensively for quality assessment of the structures and microscale damage detection in a wide range of materials in the present time. The physical experiment consists of two electronically controlled sensors. One sensor transmits a source signal that passes through the material being inspected and another sensor receives the signal after it has interacted with the material. The received signal contains the information of the state of the material, i.e., whether it is healthy or damaged and also the geometry of the damages, encoded in the received signal. The presence of the damage on the surface can be identified by studying the time-of-arrival of the originally transmitted acoustic signal through well established signal processing approaches. Yet, fine classification of the size of damage is difficult to perform using signal processing. As the material deteriorates over time, the state of damage also changes a little bit. Every time the material is inspected, it contains the information of previous damage, as well as the incremental new damage. If the difference of the size or depth of the new damage is not significant, it is almost impossible to distinguish the new and old sizes of the damage on the basis of the amplitude information of the signal. In the context of the paper, we note that the detectability of small change is determined by the wavelength of the experiment. We define ‘small change in the size of a microdamage’ as one-fifth of the wavelength of the longitudinal wave used for the measurements, which is 100 μm for the chosen experimental parameters. We note that this small change is smaller than the resolution limit for a wave, which is half wavelength. Therefore, which the microdamages of 500 μm and 600 μm are easily distinguishable from the healthy sample using simple signal processing, differentiating between these two microdamages is difficult.

We study the potential of machine learning for this problem. We show that differentiating between microdamages of sizes 500 μm and 600 μm, as well as 800 μm and 900 μm, is indeed possible by suitable selection of machine learning approach. We present two specific contributions in this respect. First, we present an evaluation of machine learning approaches using conventional feature types for this problem. We considered a variety of time-domain and frequency-domain feature types for representing the received signal and tested a variety of machine learning methods for accuracy of classification. Our results indicate that deep learning techniques do not provide specific advantage over simple machine learning algorithms. Moreover, we found that the frequency-domain feature types performed better than time-domain feature types for this problem. Second, we present new hybrid feature types that show potential for distinguishing damages with as small size difference as 100 μm.

## 2. Related Work

In the last several decades, a significant amount of effort has been dedicated to develop innovative nondestructive evaluation (NDE) techniques for damage detection on the surface and inside the ceramic components [2]. In addition to ultrasonic damage detection methods, various optical methods were also been implemented to characterize the surface and subsurface flaws in PZT ceramics. The most common optical measurements are photoacoustic microscopy, optical coherence tomography, and optical gating technique [3,4]. PZT ceramics showed a high scattering behavior at optical wavelength [4], resulting in noisy measurements. Scanning laser Doppler vibrometer has been employed for 3-dimensional visualization of acoustic wave interference with inclusions and damages in metallic plates, piezo-ceramics and piezo-crystals [5,6]. The scanning laser Doppler vibrometer experiments are expensive and require a thin reflective coating on the sample surface. In addition to the above methodologies, several ultrasonic techniques have been implemented for evaluating the health of an integrated piezoelectric sensor [7,8,9]. Rabe et al. [9] have employed atomic force acoustic microscopy to characterize elastic stiffness of the surface of PZT ceramics. The technique is restricted to quantification of surface stiffness and incapable of evaluating subsurface or bulk properties of the piezo-ceramics. Synchrotron radiation source has been explored to visualize the propagation Rayleigh wave’s on the surface of the piezoelectric crystal [10]. This technique also facilitates investigation of microscopic crystalline defects in PZT ceramics.

Over the years, we dedicated our effort to optimize the point contact excitation and detection technique to excite broadband ultrasonic waves in piezoelectric crystal and ceramics [11,12,13,14,15,16,17]. Point contact excitation and detection technique is a versatile method for generation of ultrasonic waves both in bulk modes and guided wave mode in the piezoelectric substrate. The technique leverages on the transfer of electromagnetic field to mechanical energy to excite phonon vibration in piezoelectric materials. The electro-mechanical coupling is governed by the gradient of the electric field and piezoelectric properties [17]. Here, we expand the utility of the technology for detecting damages, as well as changes in damage dimensions, through incorporation of machine learning.

Machine learning has been an important tool for processing ultrasound signals towards non-destructive evaluation and structural health monitoring. Artificial neural networks, support vector machines, and Bayesian inference models are prevalent in structural health monitoring using guided acoustic waves, as presented in a recent review [18]. Relevance vector machine [19] was proposed as a framework for processing sparse ultrasound signals. SVMs showed potential in osteoporosis screening [20]. SVMs were used for ceramic tiles’ quality classification as well [21]. K-nearest neighbor algorithm also demonstrated a good performance for the dataset in [21]. A combination of k-means algorithm and Bayesian inference was used in [22] for detecting damages in engineering materials. Bayesian inference was used for detection of damages in carbon fiber composites [23] and plate-like structures [24]. Other notable recent works that use Bayesian inference for damage detection include [25,26]. Refs. [27,28] reported using a variety of unsupervised and supervised machine learning techniques for quality control in ceramics. For ultrasonic imaging system for damage detection, a variety of unsupervised and supervised learning techniques including deep learning were compared in [29], where Fourier spectrum was found useful for feature extraction. This is closely related to our finding of power spectral density as a useful feature. Derivation of new data features from non-linear ultrasound modulation and ANN were used to detect fatigue induced cracks of size 2 mm on flat plates [30]. Sambath et al. [31] used wavelet feature with ANN for automatic damage classification of materials for non destructive inspection of structural health of the material. Kesharaju et al. [32] has used ultrasonic sensor to detect defect in sintered silicon carbide ceramic using ANN. Rizzo et al. [33] has used discrete wavelet transform based six features and ANN to detect and classify the two/three thickness(of 1 to 5 mm) of notch cut in the steel pipe. Min Meng et al. [34] also used the wavelet feature on the data extracted from the carbon fiber reinforced polymer specimens with void and delamination and then applied convolutional neural network (CNN) classifier on the data and classified the healthy and damaged data. Decision tree was used in [35] for assessing looseness of bolts. Interestingly, Ref. [35] showed that power spectral density is valuable as a feature for this problem.

We note that machine learning for point-contact excitation and detection is currently unexplored. This is a gap we intend to fill through this article. Moreover and more importantly, all the analysis used previously mostly focused on classifying healthy and damaged data. On the other hand, we cover two classification problems, (a) the conventional healthy and damaged material, and (b) the unconventional and challenging classification of damages of different dimensions. It is of significant interest and technical challenge to resolve changes of sub-wavelength scale, as an approach of early prognosis of potential material failure. In addition, we provide a study of diverse feature types and learning techniques, which allows us to holistically identify the best suited features for the challenging classification problem. We bring a feature ‘sign of slope change’, which is not used so far in the ultrasound community to the best of our knowledge. We also show that simple k-nearest neighbor (KNN) architectures are generally sufficient for classifying the damages with differences in dimension as small as 100 μm.

## 3. Experimental Technique

### 3.1. Point Contact Sensor Fabrication

**Figure 1:**
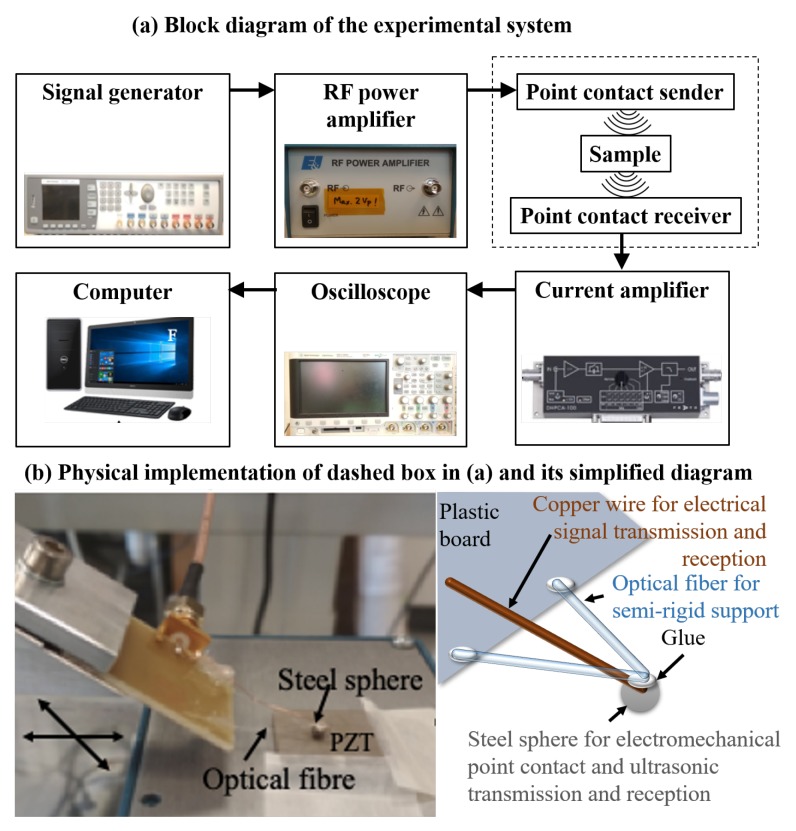
Experimental setup for point contact excitation and detection in piezoelectric (PZT) ceramics. (**a**) Block diagram of the setup. (**b**) The steel sphere works as the sender and receiver, the V-shaped structure of the optical fiber works as semi-rigid support for the steel sphere, and the PZT is the sample which may be damaged.

The technique employed is based on generation of stress wave through electro-mechanical excitation that is induced by the electric field exciting the PZT. Two small pieces (2 cm) of optical fiber were glued in such a fashion on a plastic board (6 × 4 cm${}^{2}$) that it forms a free hanging triangle shape (see Figure 1b). Below the free-suspending tip of the triangle where the fibers intersect, a steel sphere of diameter of 2.57 mm was fixed with fast epoxy glue. The steel sphere was connected with a copper wire using two component conducting silver epoxy (EPO-TEK) for excitation. A similar second probe was also constructed and placed into a metal box. Later on, both probes were placed into a vacuum oven with a temperature of 90 ${}^{\circ }$C for 2 h for solidifying the silver epoxy. In the experiments, the second probe placed in the box acts as a receiver for acquisition of the propagated ultrasonic waves. The box provides a solid surface for placing the sample stably. The first sensor, which works as sender, is scanned across a line on the sample using a translation stage. These two probes act in conjunction for simultaneous excitation and detection of bulk waves in PZT ceramics.

### 3.2. PZT Sample Preparation and Damage Creation

The characterization and detection of damage using ultrasonic waves was demonstrated on a 3 mm thick PZT ceramic. A PZT ceramics of size 20 × 20 mm${}^{2}$ was chemically etched using Ferric Chloride solution in order to remove the conducting silver electrode (Ag) from both sides. After etching the silver from both sides, the PZT was washed with distilled water and dried with Nitrogen. The PZT ceramics was washed sequentially with acetone and distilled water. Later on, the PZT samples were dried with nitrogen. After completing the experiments with healthy or reference state, a cylindrical hole of circular cross-section was drilled into the PZT to emulate the damage. A high-speed diamond drill from Dremel with drill bit diameter of 500 μm was used for this purpose. The drill hole had a depth of approximately 3 mm. After taking a line scan measurement of the damage, the diameter of the damage was increased sequentially by using drill bits of 600 μm, 800 μm, and 900 μm, respectively. The depth of the damage was retained in each case. Line scan measurements after each increment indicated the size of damage.

### 3.3. Experimental Set-Up

**Figure 2:**
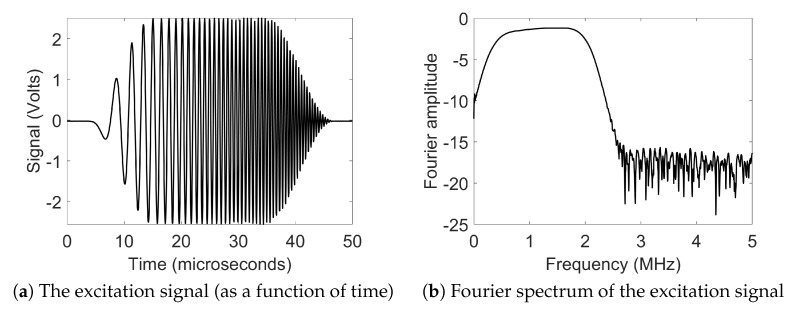
Chirp coded excitation signal used in the experiment.

Figure 1a shows the experimental setup used for performing the measurements. The experimental technique was optimized for an efficient Coloumb coupling [13] of electric field into lead zirconate titanate (PZT) ceramic, which is the material being inspected. A signal generator capable of generating user-specified arbitrary functions (Agilent 81150A) was used for the generating the excitation pulse. A chirp coded signal of width 45 μs (amplitude 5 Vpp) was excited. The excitation signal and its Fourier spectrum is shown in Figure 2. It was delivered to radio frequency amplifier (Electronics and Innovation: 403LA, New York, USA) for signal amplification. The amplified signal was then supplied to steel sphere which was gently in contact with the surface of the PZT ceramic, which then excited ultrasonic waves into the sample. The excited signal generated the bulk waves, surface wave and guided waves that propagate through the thickness of the PZT ceramic. On the opposite side of the PZT ceramics, the second probe was used for picking up the excited signal which was amplified by a trans-impedance amplifier (DHPCA-100) and delivered to the oscilloscope. This amplifier converts the current into a voltage according to an adjustable amplification factor. Finally, the voltage signal was sampled by an oscilloscope (Agilent 3024A) capable of digitizing with up to 12 bits. This oscilloscope performs averaging 256 pulse shootings, digitizes the signal, and records it onto a computer via a USB connection.

**Figure 3:**
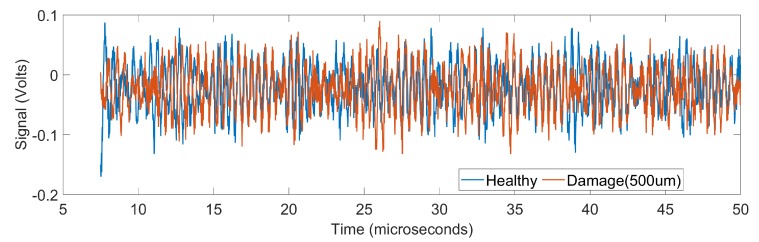
Time-domain signals corresponding to the healthy and damaged samples (damage diameter 500 μm). Here, the root mean square of the difference between the two signals is 1.024.

**Figure 4:**
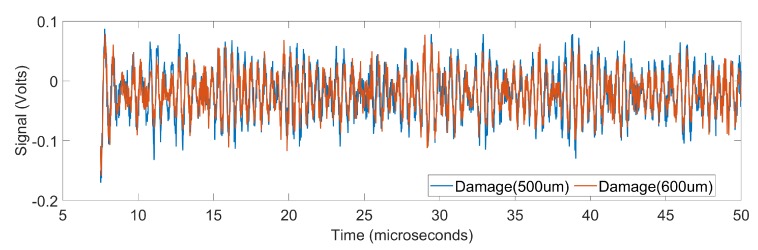
Time-domain signals corresponding to two damaged samples, one with damage of diameter 500 μm and the other with damage of diameter 600 μm. Here, the root mean square of the difference between the two signals is 0.0295.

We include comparison of the healthy and damaged data versus damaged data of two different sizes in order to illustrate the challenging nature of the problem. Figure 3 shows the amplitude of the received signal as a function of time for a healthy sample and a damaged sample. It is seen that there is a significant difference between the data of healthy and damaged samples. Figure 4 shows the amplitude of the received signal as a function of time for two damaged samples, one with diameter 500 μm and the other with diameter 600 μm. In this case, the signals almost overlap and therefore pose a challenge in classification.

## 4. Feature Extraction and Machine Learning Techniques

### 4.1. Machine Learning Techniques

We included a variety of supervised and unsupervised machine learning techniques in this study, including KNN [36], naive Bayes classifier [37], ensemble of bagged trees [38], and ensemble of subspace KNNs [39]. There variants were considered as relevant. For example, simple and weighted KNNs, naive Bayes classifier with assumption of Gaussian distribution of input variables, and naive Bayes classifier with kernel estimation were employed. In addition, deep convolution neural network (CNN) was used, whose layer details are as follows: inputLayer → convolutionLayer (1×20) → convolutionLayer (1×20) → convolutionLayer (1×10) → fullyConnectedLayer (2) → fullyConnectedLayer (2) → softmaxLayer → classificationLayer. Lastly, binary long short term memory (LSTM) [40] was used for certain features considering them as series data. The layer architecture of BLSTM used in this study is as follows: inputLayer → blstmLayer → fullyConnectedLayer (2) → softmaxLayer → classificationLayer, with 100 hidden neurons for blstmLayer. For each combination of feature type and learning approach, 10-fold cross-validation is used.

### 4.2. Feature Types

Different features in both the time and frequency domains were investigated. We derive these features using either the raw signal directly or after normalization. Normalization here is that the entire signal is shifted and scaled to span the range [0,1]. We call the signal obtained after normalization as the normalized signal.

Although the disparity in time domain signals for close datasets (such as damages of slightly different sizes) is small is time domain signals, different time-domain features may enhance the disparity of signals in such datasets. With this motivation, we considered multiple variety of time domain features, which include:

- Raw temporal signal—all the 3400 time points in the measurements were used as a single feature vector. For brevity in results, we refer to it as ‘Signal’.
- Higher order crossings (HOC) [41,42] such as used in electroencephalography (EEG) signal analysis—HOC features of the raw temporal signal of up to 50 order were used as a feature vector.
- Discrete cosine transform (DCT) [43]—we compute the DCT of the signal up to the same order as the input raw signal and retained 18.6% of DCT coefficients as the feature vector.

**Figure 5:**
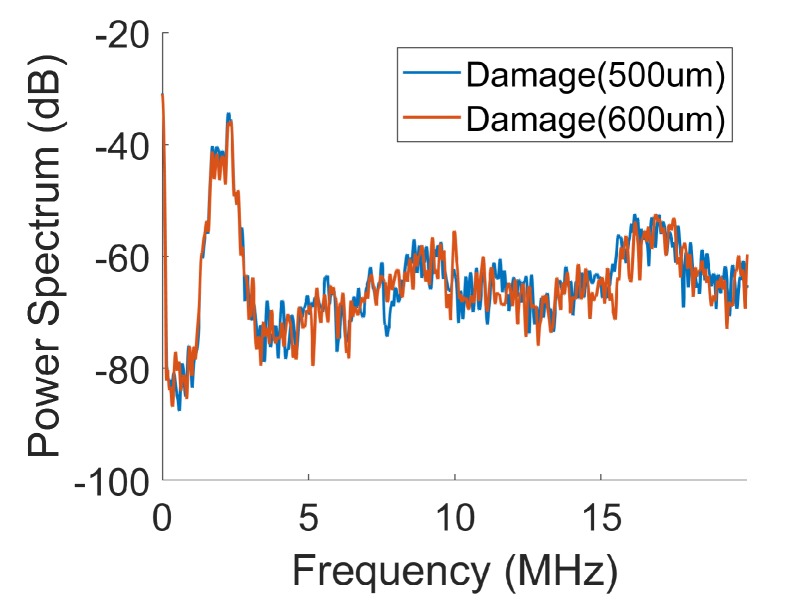
Power spectra vs. normalized frequency for data of damage (500 μm) and damage (600 μm). Here, the root mean square of the difference between the two spectra is 0.9719.

Frequency domain analysis of the data reveals a better possibility to distinguish signals from two damages of slight difference in size. To illustrate the point, we plot in Figure 5 the power spectral densities (PSDs) [44] of the two barely distinguishable signals shown in Figure 4. The differences in the power spectral densities of the two signals are more pronounced than the difference in the original signals. Root mean square of the difference in the PSDs of the two signal is 0.9719. The frequency domain features used in our study include:

- Power spectral density (PSD)—The real valued power in each Fourier component of the Fourier transform signal resulted in the PSD of the signal and was used as a feature vector. The Fourier transform of the raw signal was computed with the number of Fourier components equal to the number of samples in time domain.
- Continuous wavelet transform (CWT) [45]—the feature vector is generated using the default implementation of CWT in Matlab [44], which used Morse function and scale 1.

We noted that the HOC, DCT, and CWT features benefitted by considering principal component analysis (PCA) based dimensionality reduction for the classification problem of very similar defects. We compare the performance of original and PCA-reduced HOC, DCT, and CWT features in Table 1. The PCA features which cumulatively represented 95% of the data content was retained. The benefit of using PCA is evident for almost all the learning approaches considered. In view of this, we use PCA reduced HOC, DCT, PSD, and CWT features for the results reported hereon.

## 5. Benchmarking of the Feature Types and Learning Approaches

### 5.1. Experiment 1: Healthy Versus Damaged Samples (500 μm)

For the problem of classification of healthy versus damaged samples, the results using different feature types and different learning methods are given in Table 2. It is noticeable that the raw signal or PSD can consistently provide close to 100% accuracy for almost all learning approaches. This indicates sufficiency of the raw signal or its PSD for the simpler problem of classifying healthy and damaged sample. Interestingly, derived features, such as HOC, PSD, and CWT, perform poorer than the raw signal or PSD for this case.

We attribute this poorer performance to two interlinked factors. The first factor is the size of feature vector. The numbers of time and frequency samples, which serve as the size of feature vectors in the raw signal and PSD, respectively, are significantly larger than the number of features generated in HOC and DCT. CWT, on the other hand, generates the same number of features as the raw signal, and therefore the factor of reduced feature size is not valid for CWT. The second aspect is that PCA is applied for HOC, DCT, and CWT. While the PCA operation reduces the contribution of noise and retains statistically significant features, in the case of easily distinguishable signals, any feature lost due to PCA may still hold differentiable information. Transforming the feature space to PCA feature space was observed to be essential and it was observed that PCA features with 95% variance had more discriminatory features than raw signal or data after feature engineering for most classifiers.

### 5.2. Experiment 2: Damaged Samples of Diameters 500 μm and 600 μm

For the problem of classification of damaged samples of slightly different diameters (500 μm and 600 μm), the results using different feature types and different learning methods are given in Table 3, portion (a). The classification accuracy for any combination of feature type and learning algorithm is considerably poorer than that of experiment 1, which is not surprising for this challenging problem as explained before using Figure 3 and Figure 4. When the raw signal is used directly as feature vector for classification, the accuracy using different machine learning approaches is low, the best accuracy being 59.2% using BLSTM classifier. The use of other time domain features did not provide significant improvement, the best being 62% for DCT features combined with CNN classifier. However, frequency domain features performed significantly better. CWT provided accuracy between 70–72% for most classifiers. PSD performed better than the any other feature, reaching more than 92% for KNN and ensemble classification methods. Notably, PSD did perform poor for naive Bayes classifiers and CNN. This indicated the unsuitability of naive Bayes classifiers and the chosen CNN architecture for this challenging classification problem.

### 5.3. Experiment 3: Damaged Samples of Diameters 800 μm and 900 μm

For this classification experiment, the results using different feature types and different learning methods are given in Table 3, portion (b). The general observations of experiment 2 are valid here as well. Based on consistent observations, we conclude that

- Naive Bayes, CNN, and BLSTM architectures are not suited for this classification problem with small difference in the diameters of the damages
- PSD is an important feature type for this classification problem.

## 6. Hybrid Feature Designs for Improvement in Classification

Building upon the study in Section 5, we designed three hybrid feature designs through augmenting the PSD features as described below:

- PSD + Signal: This hybrid approach is motivated by using the temporal, as well as frequency domain, features together for classification.
- PSD + Signal + sign of slope change (SSC): Sign of slope change is an alternate way of extracting changes in temporal patterns. It was used in [46] for analyzing vibration signal. Here, we include this with the motivation of including the effect of changes in temporal patterns.
- PSD + SSC: The motivation of this hybrid feature is that it is possible that the changes in temporal pattern provide a better differentiation than the temporal signal directly.

The results of using these new hybrid features together with KNN and ensemble classifiers for experiment 2 is presented in Table 4. It is seen that the hybrid features perform better than PSD alone (reported in Table 3, portion (a)). It is also seen that the PSD + SSC hybrid feature provides classification accuracy of more than 98% using all KNN classifiers (simple, weighted, and subspace KNNs).

## 7. Discussion

Sensitivity to noise is an important aspect in any classification approach, irrespective of whether model-driven or data-driven approaches are employed. While digital denoising or filtering techniques can reduce the noise level in the raw signal, they may also filter away the discriminatory information in the signals, especially when the change in damage is small. In such situations, the differential contribution due to small change may be of the same scale as noise and the choice of noise reduction mechanism may impose if the desirable differential contribution gets contaminated or not. Noise reduction mechanisms that exploit stochasticity of noise or the noise distribution may be preferred.

Presence of noise, as well as differential signal contamination due to noise reduction technique, are both expected to impact both kind of approaches. Nevertheless, while using data driven machine learning techniques, it is important to ensure that data (including the noise) is not over-fitted during training. This may be controlled by the complexity of the model and the size of the dataset, beside the learning algorithm and the noise level. In our case, we use PCA as a mechanism of both dimensionality reduction and noise mitigation while retaining the most valuable information in the data. We opine that PCA is a suitable noise mechanism because dimensionality reduction based on eigenvalues removes the least correlated portion of data which is likely to be noise since the noise would be more uncorrelated than the differential signal. These aspects provide model simplification, as well as noise sensitivity. It is therefore not surprising that simple models such as KNN provide superior overall performance than other more sophisticated methods. Nonetheless, noise sensitivity of a machine learning approach for a selected application and experimental setup is indeed interesting. This, however, requires an extensive study with good control of signal to noise ratio and qualification of noise sources. Therefore, we relegate this study to the future. In that study, it will also be interesting to compare the performances of model-driven and data-driven models for the smallest distinguishable change at a particular signal to noise ratio.

## 8. Conclusions

This paper considers the challenging problem of classifying damages with 100 μm difference in diameters. Beside studying conventional features and classifiers, this article provides specific insights into suitable approaches for this problem. The first insight is that CNN and LSTM architectures may not be the best suited for this problem. Power spectral densities of the received acoustic signals are the key features that make this problem approachable. However, the newly proposed hybrid feature that includes PSD, as well as SSC (indicating the changes in the temporal pattern), provides up to 98% classification accuracy using a variety of KNN classifiers. Based on the observations, we infer that the classifier is not the bottleneck for this problem and simple KNN architecture may suffice in practice. However, the key ingress in this challenging problem is through Fourier domain analysis combined with changes in temporal patterns.

## Figures and Tables

**Table 1:** Classification accuracy for the classification of damages of diameters 500 μm and 600 μm without using principal component analysis (PCA)-based dimensionality reduction (ori) and with using PCA-based dimensionality reduction. KNN = k-nearest neighbor; HOC = higher order crossings; DCT = discrete cosine transform.

	HOC		DCT		CWT	
Classifier	ori	PCA	ori	PCA	ori	PCA
KNN (simple)	33.3%	58%	42.8%	59.2%	47.9%	70.8%
KNN (weighted)	20.8%	54%	56.4%	59.2%	70.8%	72%
Naive Bayes (Gaussian)	20.8%	58.3%	54%	54%	54.2%	54.2%
Naive Bayes (Kernel)	20.8%	58.3%	54%	54%	64.6%	70.8%
Ensemble (bagged trees)	20.8%	51.2%	46%	56%	68.8%	70.8%
Ensemble (subspace KNNs)	20.8%	55.2%	56.4%	59.2%	68.8%	70.8%

**Table 2:** Classification accuracy for healthy versus damaged sample (diameter 500 μm). Accuracy of better than 90% is shown in grey background. (CNN = convolutional neural network; BLSTM = bidirectional long short term memory; CWT = continuous wavelet transform).

Classifier	Signal	HOC	DCT	PSD	CWT
KNN (simple)	100%	91.7%	95.4%	99.9%	96.8%
KNN (weighted)	100%	95.8%	95.4%	100%	93.8%
Naive Bayes (Gaussian)	100%	91.7%	92.4%	97.9%	54.2%
Naive Bayes (Kernel)	100%	91.7%	92.4%	99.8%	79.2%
Ensemble (bagged trees)	100%	87.5%	94.2%	99.8%	95.8%
Ensemble (Subspace KNN)	100%	91.7%	95.4%	99.9%	95.8%
CNN	99.2%	96.8%	94.5%	99.4%	96.2%
BLSTM	95.1%	94.4%	-	-	-

**Table 3:** Classification accuracy for damaged samples. Accuracy of better than 90% is shown in grey background.

	(a) Diameters 500 μm and 600 μm					(b) Diameters 800 μm and 900 μm				
Classifier	Signal	HOC	DCT	PSD	CWT	Signal	HOC	DCT	PSD	CWT
KNN (simple)	52.6%	58%	59.2%	94.7%	70.8%	58%	73%	55.7%	95.5%	72.9%
KNN (weighted)	52.8%	54%	59.2%	94.8%	72%	58%	64.3%	52.4%	95.4%	72.9%
Naive Bayes (Gaussian)	51.3%	58.3%	54%	50%	54.2%	52%	57.1%	46%	50.5%	54.2%
Naive Bayes (Kernel)	56.5%	58.3%	54%	53.5%	70.8%	52%	46.4%	46%	53.3%	70.8%
Ensemble (bagged trees)	55.3%	51.2%	56%	92.8%	70.8%	51%	73%	51%	93.6%	72.9%
Ensemble (Subspace KNN)	52.7%	55.2%	59.2%	94.7%	70.8%	58%	73%	55.7%	95.5%	72.9%
CNN	58%	55.9%	62%	64%	72.8%	59.6%	75.2%	61%	68%	73.4%
BLSTM	59.2%	60%	-	-	-	57%	81.2%	-	-	-

**Table 4:** Classification accuracy for damaged samples of 500 μm and 600 μm using hybrid parameters followed by PCA with 95% variance. Accuracy of better than 90% is shown in grey background.

Classifier	PSD + Signal	PSD + Signal + SSC	PSD + SSC
KNN (simple)	96.6%	97.5%	98.2%
KNN (weighted)	96.6%	97.5%	98.2%
Ensemble (bagged trees)	94.9%	95.2%	96.4%
Ensemble (Subspace KNN)	94.6%	97.5%	98.2%
